# Toxicity and Metabolism of Layered Double Hydroxide Intercalated with Levodopa in a Parkinson’s Disease Model

**DOI:** 10.3390/ijms15045916

**Published:** 2014-04-09

**Authors:** Aminu Umar Kura, Nooraini Mohd Ain, Mohd Zobir Hussein, Sharida Fakurazi, Samer Hasan Hussein-Al-Ali

**Affiliations:** 1 Laboratory of Vaccine and Immunotherapeutic, Institute of Bioscience, Universiti Putra Malaysia, Selangor 43400, Malaysia; E-Mail: aminuukura@yahoo.com; 2 UPM MAKNA Cancer Research Laboratory, Institute of Bioscience, Universiti Putra Malaysia, Selangor 43400, Malaysia; E-Mail: aini_aini78@yahoo.com; 3 Materials Synthesis and Characterization Laboratory, Institute of Advanced Technology (ITMA), Universiti Putra Malaysia, Selangor 43400, Malaysia; E-Mail: mzobir@science.upm.edu.my; 4 Faculty of Medicine and Health Science, Pharmacology Unit, Universiti Putra Malaysia, Selangor 43400, Malaysia; 5 Laboratory of Molecular Biomedicine, Institute of Bioscience, Universiti Putra Malaysia, Selangor 43400, Malaysia; E-Mail: sameralali72@yahoo.com; 6 Faculty of pharmacy, Isra’a University, P.O. Box 22, Amman 11622, Jordan

**Keywords:** zinc-aluminum, nanocomposite, cytotoxicity, PC 12, levodopa, LDH

## Abstract

Layered hydroxide nanoparticles are generally biocompatible, and less toxic than most inorganic nanoparticles, making them an acceptable alternative drug delivery system. Due to growing concern over animal welfare and the expense of *in vivo* experiments both the public and the government are interested to find alternatives to animal testing. The toxicity potential of zinc aluminum layered hydroxide (ZAL) nanocomposite containing anti-Parkinsonian agent may be determined using a PC 12 cell model. ZAL nanocomposite demonstrated a decreased cytotoxic effect when compared to levodopa on PC12 cells with more than 80% cell viability at 100 μg/mL compared to less than 20% cell viability in a direct levodopa exposure. Neither levodopa-loaded nanocomposite nor the un-intercalated nanocomposite disturbed the cytoskeletal structure of the neurogenic cells at their *IC*_50_ concentration. Levodopa metabolite (HVA) released from the nanocomposite demonstrated the slow sustained and controlled release character of layered hydroxide nanoparticles unlike the burst uptake and release system shown with pure levodopa treatment.

## 1. Introduction

Nanoparticles are nanometer (nm) sized substances whose size results in unique properties and leads to improvement in the field of drug delivery. However, their potential adverse health effect is of concern, especially to the users [1]. The term nanotoxicity was coined in 2004, referring to the study of the potential toxicity of nanoparticles on biological and ecological systems; it arose due to concern over the growing field of nanotechnology and the potential health effects of nano materials [2]. The low solubility or insoluble type nanomaterial constitutes the greater concern, since they are capable of passing through various defense systems due to their small size [2].

Layered double hydroxide (LDH) is a form of nanomaterial, commonly synthesized using either ion exchange or a co-precipitation method. These particles are less toxic than most other nano-carriers, they yield products that are tissue friendly under physiological conditions [3], and their general biocompatible nature makes them an acceptable alternative drug delivery system [4]. Structurally, there exists a weak bond between the interlayer anions and hydroxides sheets of LDH allowing for exchange of anions, a characteristic feature of LDH [5]. Negatively charged drugs like levodopa, when intercalated between the two-nano layer sheets will gain extra stability due to the interaction of the two cationic brucites (interlayer sheets) with the anionic negatively charged drug. Unlike the anionic drug, neutral hybrids can enter through the negatively charged cell surface without repulsion and once inside, a cell lysosomal enzyme will break it down to release the drug [6]. Meanwhile, levodopa is still the standard treatment of choice in the symptomatic management of Parkinson’s disease and in slowing down disease progression [7]; however, there is an increased concern with the ever growing evidence of its neurotoxic tendencies, demonstrated by both cell and animal model studies [7]. This neurotoxicity is believed to originate from the levodopa itself and its metabolites, especially evident in neuronal cell lines [7]. Oxidative stress induction of the cells by levodopa or its metabolites leads to cell demise via apoptosis [7]. Currently, synthesis of nano delivery systems containing levodopa is increasing [8–10]. These new delivery systems may likely reduce the pulsatile stimulation of dopaminergic neurons and will deliver levodopa to the brain in a sustained and controlled release fashion, thus, reducing the risk of levodopa-induced dyskinesia and other related side effects.

Our previous manuscript [8] detailed the synthesis of zinc aluminum levodopa nanocomposite (ZAL), where a co-precipitation method was used to intercalate levodopa between the two-nano sheets. X-ray diffraction (X-RD), Fourier transform infrared spectroscopy (FTIR) and thermogravimetric analysis (TGA) were used to prove intercalation and thermal stability of the new nanocomposite. Pseudo-second order kinetics was demonstrated to govern the release of the 16% loaded levodopa from the nanocomposite in a pH-dependent fashion. Among the interesting findings in that study was the decreased cytotoxicity shown by ZAL compared to pure levodopa on a fibroblast cell line [8].

Thus, here we aimed to assess further possible toxicity and the ability of ZAL to alter cell morphology under toxic environments. In addition, we also attempted to study the *in vitro* drug delivery and metabolism by a Parkinson’s disease cell model following incubation with the nano-carrier containing levodopa. PC12 cells are derived from rat, with a capability to produce and excrete neurotransmitters, especially after full differentiation into a complete dopaminergic cell by nerve growth factor (NGF) [11]. The cell line can be used as an *in vitro* drug metabolism model of dopamine due to the presence of monoamine oxidases (MAOs) A and B, flavo-enzymes that catalyze the oxidative deamination of biogenic amines including neurotransmitters [11].

## 2. Results and Discussion

### 2.1. Cell Viability Study

**Figure 1.**
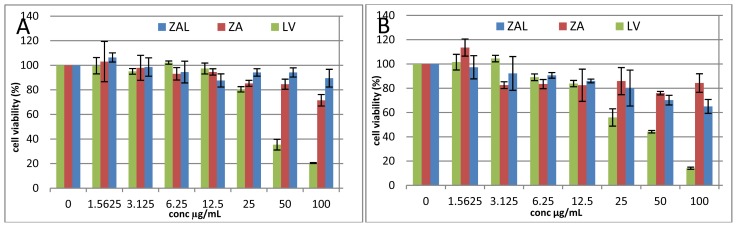
Dose and time dependent viability changes of PC12 cells at 48 h (**A**) and 72 h (**B**) post exposure to levodopa (LV), zinc aluminum nanocomposite (ZA) and zinc aluminum-levodopa nanocomposite (ZAL). The figures showed cell viability decreases with increase in dose and time, more with pristine levodopa than the corresponding nano delivery system. *IC*_50_ of ZAL, ZA and LV are 178.67 ± 2.6, 154.09 ± 3.4 and 49.37 ± 1.2 μg/mL, respectively. There are no significant differences within or between the groups and the control as tested by one-way ANOVA (*p* > 0.05).

MTT [3-(4,5-dimethylthiazol-2-yl)-2,5-diphenyltetrazolium) assay was conducted to evaluate the cytotoxicity of zinc aluminum nanomaterial containing levodopa on PC12 cell line as an *in vitro* model for Parkinson’s disease, a widely used cell line with neuronal characteristics [8,12,13]. The ZAL, ZA nano delivery systems with and without levodopa (respectively) and the pristine levodopa (LV) showed dose and time dependent cell viability effects. Viability above 80% after 48 h incubation at 100 μg/mL of both ZAL and ZA was demonstrated. Pristine levodopa on the other hand decreases the viability, to less than 30% at 100 μg/mL (Figure 1A). The decrease in viability was seen more with increasing doses after 72 h (Figure 1B). Hence, the effects of the treatments are a dose and time dependent.

Levodopa causes greater cell death than ZAL nanocomposite, while ZA nanocomposite causes a negligible effect on viability when compared dose for dose, with either ZAL or LV (Figure 1A,B). This is in agreement with previously reported studies involving pristine levodopa on the PC12 cell line that reported toxicity of the former to the later via free radical generation [14]. The effect of sustained release levodopa from the carrier on the cell line, but not the carrier itself causes cell viability decrease in ZAL-treated cells, which is not seen in ZA-treated cells. In our earlier study, zinc aluminum layered hydroxide exposure to another type of normal cell line (3T3 mouse fibroblast) at 100 μg/mL did not significantly affect the cell viability after 72 h [8]. Zinc aluminum nano-layer intercalated with different drugs demonstrated similar positive cell viability effects on tested cell lines within these dose ranges [15,16]. Cytotoxicity findings due to LDH exposure varied greatly according to tested cell type, with LDH of magnesium nitrate having slightly less toxicity than that of zinc nitrates. However, when comparing the toxicity of LDH of both zinc and magnesium nitrates to that of iron oxide, carbon nanotubes, titanium oxide, silica and other inorganic nanoparticle, LDH is the least toxic among them [3].

**Figure 2.**
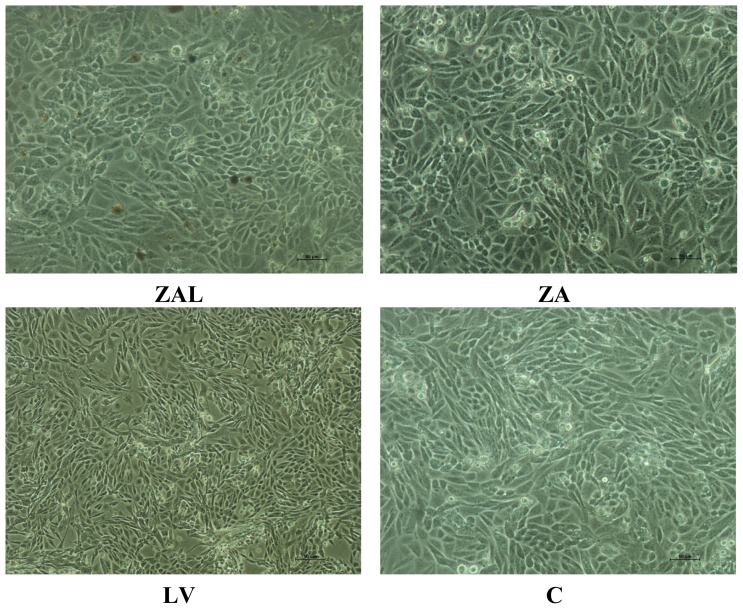
PC12 cells’ morphological appearance on an inverted microscope at 20 magnifications after treatment with *IC*_50_ values obtained from MTT proliferation assay and viewed after 72 h after treatment. ZAL, ZA, LV and C are the cells treated with zinc aluminum-levodopa nanoparticle, zinc aluminum nanoparticle, levodopa and untreated (control) respectively. No obvious changes noted between 24, 48 and 72 h post treatment. Cells from the three treated groups maintained an ovoid to spherical shape, similar to those in the control group. Scale bar = 50 μm.

**Figure 3.**
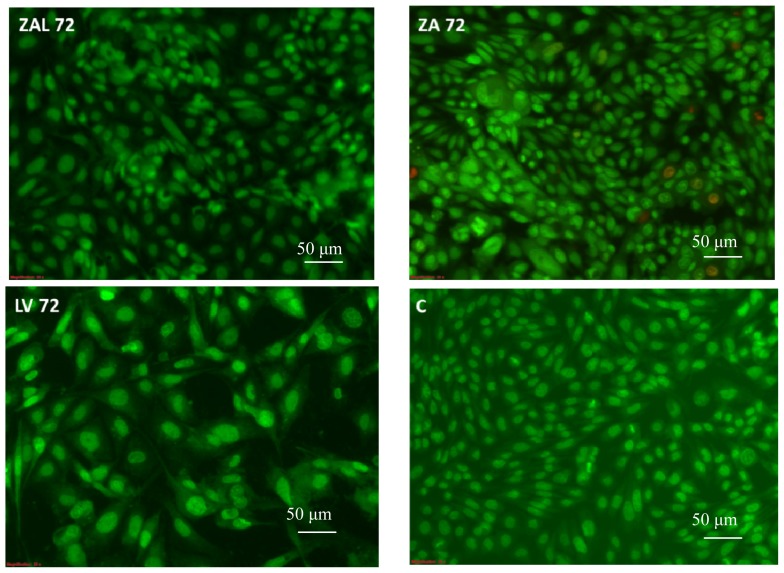
PC12 cell’s morphological appearance on a fluorescence microscope at ×20 magnification after treatment, with *IC*_50_ values obtained from the proliferation assay and stained with acridine orange/propiodine iodide. ZAL, ZA, LV and C are the cells treated with zinc aluminum-levodopa nanoparticle, zinc aluminum nanoparticle, levodopa and untreated (control) after 72 h. These are the viable cells (stained by the acridine orange only) maintaining an ovoid to spherical shape. Scale bar = 50 μm.

The cells showed an ovoid to spherical normal shape after exposure to an *IC*_50_ concentration of ZAL, ZA and LV obtained from the above MTT-proliferation assay in both pre-stain and post-stain study (Figures 2 and 3). The spherical shape seen in the treated wells was comparably similar to the control untreated wells (C). Dead (detached, loose) cells (Figure 2) have a round shape as shown on the inverted microscope. An *IC*_50_ concentration has the ability to kill fifty percent of the exposed cell, yet, the remaining surviving cells maintain a normal physiological shape. In a related study by another group, they reported morphological changes to PC12 cells exposed to a nanoparticle (iron oxide nanoparticle) over six days [17]. The effect on cell morphology was found to be dose-dependent, affecting only the neurites at the lower dose and the main cell membrane at the higher dose. Nanoparticles induce injury to cells and tissues through several mechanisms, among which is cell membrane cytoskeleton disruption targeting of actin protein [17]. Changes in cell morphology with cell surface bleb formation may rupture the cell and cause the release of intracellular chemical substances, some of which are toxic to neighboring cells and tissues. The cell’s cytoskeleton, of which actin is a key component, plays a central role in forming the cell shape, motility, division, tissue organization, and other biologically important processes [17].

**Figure 4.**
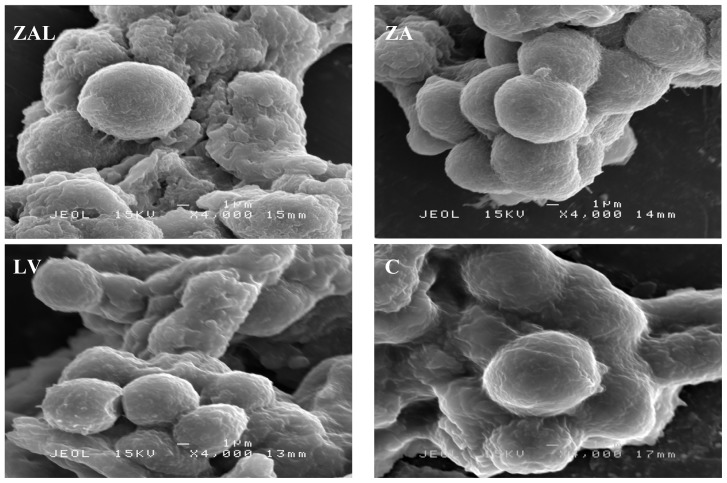
PC12 cell’s morphological appearance on scanning electron microscope (SEM) at ×4000 magnification after treatment with *IC*_50_ values obtained from MTT-proliferation assay and viewed 72 h after treatment. ZAL, ZA, LV and C are the cells treated with zinc aluminum-levodopa nanoparticle, zinc aluminum nanoparticle, levodopa and control (untreated cells). The cells from treated samples ZAL, ZA and LV maintained a spherical appearance similar to that of cells from control sample C. Scale bar = 1 μm.

Undifferentiated PC12 cells are spherical in shape and without neurites [18], which is much like the structure shown in “C” (Figure 4), obtained from control cells after 72 h of seeding in the media without any levodopa or nanocomposite. However, the morphology of treated cells with ZAL, ZA and LV were comparatively similar to that of control, unaltered despite exposure to the nanodelivery systems and pure levodopa over 72 h periods. In a related study, hepatocyte exposure to acetaminophen causes significant morphological changes as seen by both light and electron microscope with internal rearrangement of mitochondria and other organelles as a sign of toxicity [19].

Layered hydroxide uptake into cells was shown to be via an energy-dependent clathrin mediated endocytotic pathway into the cytoplasm of cells [20,21]. Between the pH values 2.2 and 8.8, levodopa exists in a zwitterionic form where the carboxylic group is deprotonated and appeared positively charged on the ammonia group (Figure 1) [22]. Synthesis of ZAL nanocomposite was done between the pH value 3–7 [8], while the release and metabolism study here was done between pH 7–7.2.

**Figure 5.**
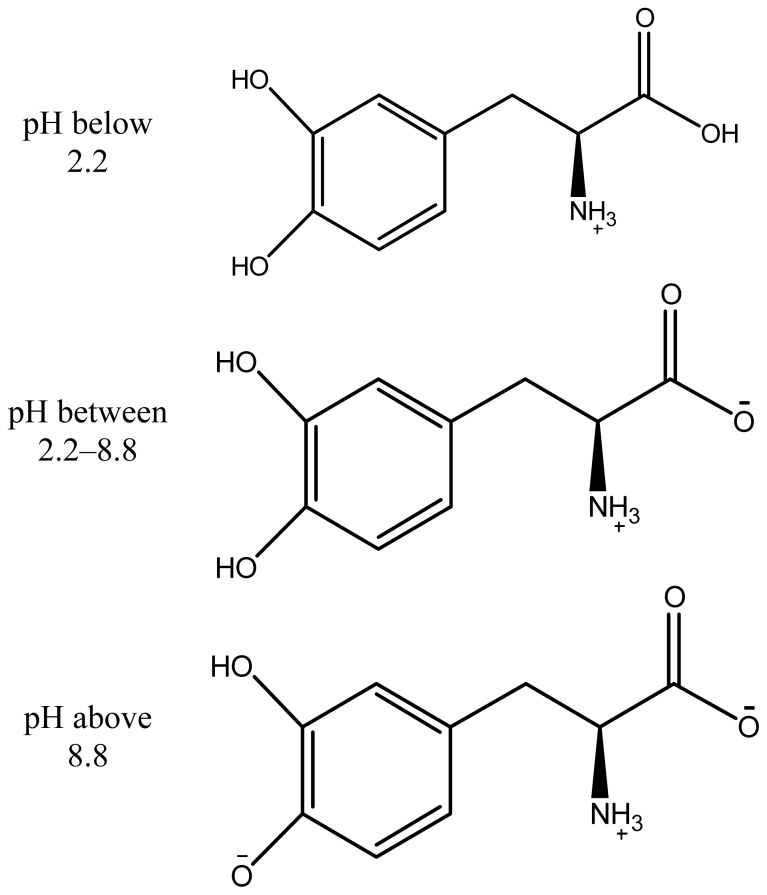
Structure of levodopa modification at different pH values.

In addition, the levodopa below pH 2.2 has fully protonated and exists as a + 1 cation; whereas above 8.8, one hydroxyl group deprotonated to form a net charge of −1 [22,23]. We previously mentioned that ZAL nanocomposite has a positively charged surface [8], and on the basis of the speciation of levodopa seen in Figure 5, levodopa may interact with the LDH surface via the negatively charged COO^−^ functional group [8]. Therefore the release from the layered sheet is usually via ion exchange and is pH-dependent in a sustained release fashion [24].

**Figure 6.**
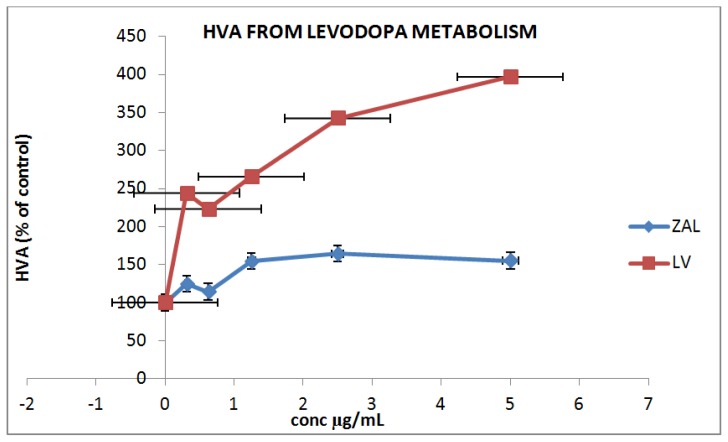
Dopamine metabolite (HVA) release from pure levodopa (LV) and levodopa intercalated in zinc aluminum nano delivery system (ZAL). There is a statistically significant difference between the three groups, *p* < 0.01 (*i.e.*, Control, ZAL and LV) as tested by one-way ANOVA. A further test using Dunett’s *post-hoc* test shows a significant difference between ZAL and LV, also between LV and control, but not between ZAL and control.

Drug uptake and its subsequent metabolism constitute an important component of pharmacokinetics [25]. In the case of levodopa, homovalinic acid (HVA) is one of the metabolites formed after its uptake and metabolism by the cells. Here, we analyzed the production of HVA of a differentiated PC12 cell line following treatment with different concentrations of LDH intercalated with levodopa to compare with the effect seen with pure levodopa. The metabolite production seen from both treatments were found to be in a dose dependent pattern with levodopa having a higher production of the HVA metabolite compared to the LDH nano delivery system. Unlike pure levodopa, the nano delivery system has a slow, sustained release property that may last for days (Figure 6). In our previous study, we reported that the release of levodopa from this delivery system under two different pH values (pH 4 and 7) lasted more than 72 h each [8]. This may explain the lower level of HVA metabolite production seen when LDH nanocomposite was incubated with PC12 cells when compared to incubation with pure levodopa. Nevertheless, cellular uptake, drug release and the metabolism of levodopa from the nanocomposite on a Parkinson’s model were indirectly demonstrated by the presence of metabolites (HVA) release in this experiment.

## 3. Materials and Methods

### 3.1. Cell Culture

We obtained a rat neuronal cell line (PC12) from the American Type Culture Collection ((ATCC), Manassas, VA, USA). RPMI 1640 media, supplemented with 10% fetal bovine serum, 100 units/mL penicillin, and 100 mg/mL streptomycin used throughout the experiment, cells were cultured under a humidified atmosphere (5% CO_2_ plus 95% air) at 37 °C. All other reagents used were of analytical grade and used without further purification.

### 3.2. Preparation of Nanoparticles for Viability Assay

Freshly prepared zinc aluminum-levodopa nanocomposite (ZAL) and zinc aluminum nanocomposite (ZA) were used to treat cells. They were dispersed in PBS solution, and to ensure uniform suspension, nanoparticle stock suspensions of 10 mg/mL were made through sonication for 5 min and culture medium was used to obtain the desired concentration via serial dilution. To disperse our nanoparticles further, vortex agitation was applied for 60 s before every use.

### 3.3. Cell Viability Study

The mitochondrial functions of viable cells were measured in MTT assay, PC12 cells were seeded into a 96-well plate at a density of 1.0 × 10^5^ cells/mL and a volume of 100 μL. After an overnight incubation in complete medium for attachment, cells were exposed to nanoparticles (ZAL and ZA) and pure levodopa containing media. Forty-eight and 72 h post treatment with different concentrations (0–100 μg/mL) of nanocomposite and pure levodopa, cells were analyzed for the toxicity potentials of levodopa and nanocomposite.

The assay was done according to Mossman’s work [26] with few modifications as described in our previous work [8]. Principally, the MTT assay is dependent on the reduction of the tetrazolium salt (3-(4,5-dimethylthazol-2-yl)-2,5-diphenyl tetrazolium bromide) by the mitochondrial dehydrogenase of only viable cells to form a blue formazan product [26]. In brief, media from treated cells and the control group was carefully discarded; this is followed by a washing step using PBS at 100 μL/well. To each well 200 μL of 0.5% *w*/*v* MTT dissolved in media was added, and the plates were further incubated in a humidified incubator for 2 h. This is to allow for the conversion of tetrazolium salt by the mitochondrial dehydrogenase enzymes of the viable cell to formazan crystals. Dimethyl sulfoxide (DMSO), a detergent, was added to each well and mixed vigorously in order to dissolve formazan crystals. In order to dissolve the formazan thoroughly and to distribute it evenly, the plate was kept on a shaker in the dark for 15 min before taking the optical density reading at 570 nm and background 630 nm. This directly correlates with viable cell quantity.

Cytotoxicity calculated as:

Cell viability (%)=[Average] test/[Average] control×100%

### 3.4. Surface Morphological Changes of PC12 Cells Due to Exposure to Nanocomposites or Pure Levodopa

#### 3.4.1. Microscopic Study

To observe the morphological changes due to exposure to nanocomposite and pure levodopa, we treated PC12 neuronal cells with the obtained *IC*_50_ values from MTT assay. Using an inverted microscope (Olympus Corporation, Shinjiku-ku, Tokyo, Japan) the cells were viewed 72 h post treatment at 20 magnifications. Untreated cells were used as a control to compare with the treated cells for any change in morphology.

Cell exposed to stress due to nutrient deprivation or toxic drugs die through one of these two methods namely necrosis or apoptosis. The viable (green intact cells), apoptotic (green shrinking cells with condensed of fragmented nucleus), and necrotic (red cells) were the morphological changes expected under fluorescence microscope after staining with acridine orange and propiodium iodide. Propidium Iodide (PI) intercalates into double-stranded nucleic acids by penetrating the cell membrane of dying cells, but is excluded by viable cells. Acridine orange (AO) on the other hands stain viable cells due to its ability to penetrate intact cell membranes of viable cells.

The cells were stained with these two dyes after viewing under the inverted microscope. To each well 5 μL of dye mixture containing 100 μg/L AO and 100 μg/L PI in PBS added and cells were visualized immediately under a fluorescent microscope for the above-mentioned morphological changes at 20× magnifications within 30 min.

#### 3.4.2. Scanning Electron Microscope (SEM) Analysis

Cells treated with nanocomposite, levodopa and the control were fixed in 4% glutaraldehyde for 4 h at room temperature. Secondary fixation follows in 1% osmium tetroxide (aqueous) pH 7.4 for 1 h at room temperature after three washes in 0.1 M cacodylate buffer (pH 7.4) for 10 min each. Samples were washed again three times in 0.1 M cacodylate buffer before serially dehydrating in ascending concentrations of acetone 35%, 50%, 75%, 95% and 100%, each for 10 min except the 100% dehydration that was done three times lasting 15 min each. Finally, the samples were dried via an automated process and mounted onto the metal stub with double -sided carbon tape. The specimens were viewed at 4000 magnifications on a JEOL SEM (JEOL USA, Peabody, MA, USA).

#### 3.4.3. *In Vitro* Drug Metabolism and Uptake

The dopaminergic cell (PC 12) were grown in complete RPMI-1640 medium as described earlier. Cells were forced to differentiate to a fully functioning neural cell by changing the media to a NGF containing media. About 4 × 106 cells/mL were cultured in a new flask and the medium was changed to new medium containing NGF (NGF-7S, Sigma-Aldrich, Inc., St. Louis, MO, USA) at 100 ng/mL. Media was changed every two days and the cells were allowed to reach their full growth in seven days, then, drug metabolism study was carried out. PC12 cells usually extend their neurites after differentiation and eventually become electrically excitable, efficiently responding to exogenously applied neurotransmitters [27]. The differentiated cells were cultured on a 24-well plate at a density of 1 × 10^6^ and allowed to attach overnight. Media was discarded and new media containing levodopa, and ZAL nanocomposite in serial dilution was added to the cells; a dose range of 0–5 μg/L was used, where zero concentration was the control treatment. To determine the uptake and metabolism of levodopa by the cell line after 24 h treatment, the media containing drug treatments were discarded and cells harvested mechanically. Several freeze-thawing cycles inside ice cold PBS was applied and using a homogenizer pestle, the cells were disrupted to get a good lysate. The lysate was centrifuged at 15,000 rpm for 15 min at 4 °C and the supernatant obtained was used to detect the HVA level based on the instruction provided by the manufacturer of CUSABIO rat homovalinic acid (HVA) assay kit (Wuhan, Hubei, China).

## 4. Conclusions

The synthesized layered hydroxide nanocomposite containing the anti-parkinsonian agent levodopa shows the minimal toxicity potential of a PC12 cell Parkinson’s disease model in a dose and time-dependent pattern compared to pure pristine levodopa. Un-intercalated layered hydroxide nanoparticle had no toxic effect of the cell line, within the concentrations used. The disease model basic cytoskeletal structure remained unaltered in the presence of this nano delivery system at the selected high dose and its uptake, release and metabolism followed more controlled, sustained and continuous patterns, making it a possible alternative choice for a nano delivery system for chronic Parkinson’s disease treatment rather than conventional levodopa.
